# Silicone wristbands detect individuals' pesticide exposures in West Africa

**DOI:** 10.1098/rsos.160433

**Published:** 2016-08-17

**Authors:** Carey E. Donald, Richard P. Scott, Kathy L. Blaustein, Mary L. Halbleib, Makhfousse Sarr, Paul C. Jepson, Kim A. Anderson

**Affiliations:** 1 Food Safety and Environmental Stewardship Program, Environmental and Molecular Toxicology, Oregon State University, ALS 1007, Corvallis, OR 97330, USA; 2 Integrated Plant Protection Center, Oregon State University, 2040 Cordley Hall, Corvallis OR 97330, USA; 3 United Nations Food and Agriculture Organization, 15 rue Calmette x Assane Ndoye, BP 3300 Dakar, Senegal

**Keywords:** Senegal, agriculture, exposome, exposure, passive sampling device

## Abstract

We detected between 2 and 10 pesticides per person with novel sampling devices worn by 35 participants who were actively engaged in farming in Diender, Senegal. Participants were recruited to wear silicone wristbands for each of two separate periods of up to 5 days. Pesticide exposure profiles were highly individualized with only limited associations with demographic data. Using a 63-pesticide dual-column gas chromatography–electron capture detector (GC-ECD) method, we detected pyrethoid insecticides most frequently, followed by organophosphate pesticides which have been linked to adverse health outcomes. This work provides the first report of individualized exposure profiles among smallholder farmers in West Africa, where logistical and practical constraints have prevented the use of more traditional approaches to exposure assessment in the past. The wristbands and associated analytical method enabled detection of a broad range of agricultural, domestic, legacy and current-use pesticides, including esfenvalerate, cypermethrin, lindane, DDT and chlorpyrifos. Participants reported the use of 13 pesticide active ingredients while wearing wristbands. All six of the pesticides that were both reportedly used and included in the analytical method were detected in at least one wristband. An additional 19 pesticide compounds were detected beyond those that were reported to be in use, highlighting the importance of measuring exposure in addition to collecting surveys and self-reported use records. The wristband method is a candidate for more widespread use in pesticide exposure and health monitoring, and in the development of evidence-based policies for human health protection in an area where food security concerns are likely to intensify agricultural production and pesticide use in the near future.

## 1. Introduction

Increases in both global population and *per capita* food consumption require sustainable intensification of agricultural production in order to increase the food supply while minimizing additional impacts on the environment [1,2]. Global pesticide production is estimated to increase 1.7-fold between 2001 and 2020 [3] in response to this anticipated expansion in production. Climate change is also expected to contribute to the food shortage burden and exacerbate pesticide use, particularly in the developing world [4]. While only 2–5% of global pesticide use is in Africa, health risks to African farmers are disproportionately high because of poor handling practices, uneconomical use patterns, lack of knowledge about pesticide toxicity and exposure pathways, and the availability of pesticides banned or unauthorized in developed countries [5–7].

Given the high mammalian toxicity of many of the pesticides used in Africa, effective strategies are necessary to quantify individual risks to farmers. Using data from surveys administered to 1704 farming family members in 19 villages across five West African countries, Jepson *et al.* [6] modelled pesticide use practices, and identified substantial human and ecological health risks. Levels of risk varied considerably among villages within the five studied countries. Although there is very low residual uncertainty associated with these pesticide risks to human health and the environment throughout West Africa [8], direct measurements of personal exposure have not yet been published. The lack of direct analysis of human and environmental exposures is a result of low capacity for chemical analysis in the region, and the limited suitability of many of the available methods of monitoring [6,9].

Anderson *et al.* [9] employed passive sampling devices to determine the freely dissolved fraction of pesticides in West African irrigation water used not only for agriculture, but also for drinking, bathing and washing. Passive sampling methods have been used extensively in recent decades and mimic the passive uptake of freely dissolved or vapour-phase organic contaminants in water or air [9–12]. Human occupational exposure profiles for pesticides have traditionally been obtained through obtrusive active sampling methods, including urine collection [13,14], hand-wash samples [14–16], breathing zone air pumps [16–18] or whole body dosimetry [17,18]. Passive sampling approaches are less burdensome for participants, and commonly consist of dermal patches [15,19].

Recently, O'Connell *et al.* [20] demonstrated an adaptation of passive sampling technology with an easy-to-wear silicone wristband, allowing individualized exposure characterization. Because the wristband material non-specifically sequesters non-polar and semi-polar contaminants, we hypothesized that wristbands could also be used to assess pesticide exposure in farm workers. This investigation represents a first use of this technology in Africa, and also the first case of direct measurement of the pesticide residues to which these farmers may be exposed. We expanded an existing semi-quantitative chemical screening analysis [20] to accommodate quantitative analysis of 63 pesticides with an optimized method for gas chromatography using electron capture detection (GC-ECD) that achieved detection limits as low as 0.046 ng g^−1^ wristband.

This work was undertaken in Diender, a rural farming community in the Niayes region of Western Senegal. Farming in this and other similar areas is a family task in which men, women, children and even infants are present in the field [6]. Community members demonstrated interest in decreasing the risks associated with pesticide use following a farmer education programme in early 2014 [8] and agreed to participate in this investigation.

The objectives of this work were to examine the utility of passive sampling technology to detect and measure a wide range of pesticides, to quantify pesticide exposure profiles among individual members of a farming community, and to identify potential demographic risk factors. Individual pesticide exposure information provides valuable feedback to Diender farmers; it is intended to enable more informed decision making about pesticide use, and contribute evidence of the degree to which farmers are directly exposed to toxic chemicals. This evidence has the potential to inform policy, and the methodology reported here could serve as the basis for widespread long-term monitoring of pesticide exposure, which could help to underpin sound chemical management [21].

## 2. Material and methods

### 2.1. Material

Sixty-three target pesticides and related compounds were analysed in wristbands (electronic supplementary material, table S1). Three extraction surrogate standards; tetrachloro-meta-xylene (TCMX), PCB-100 and PCB-209; and an internal standard *p,p′*-dibromooctofluorobiphenyl were used. All standards were of purity 97% or greater and purchased from Accustandard (New Haven, Conn., USA). Ethyl acetate solvent was Optima grade or equivalent. Glassware and other laboratory equipment were solvent-rinsed and baked before use. Two sizes of silicone wristbands were purchased from an online distributer (https://24hourwristbands.com; large: 4.8 ± 0.1 g; small: 4.3 ± 0.1 g; both sizes width = 0.5 inch).

### 2.2. Population sample and data collection

Thirty-five men and women from farming families in Diender, Senegal were recruited in November 2014. All research activities were granted prior approval by Oregon State University's Institutional Review Board (No. 6479). Children who are active in the field, or accompanied parents could be included with parental consent, as long they also consented. After providing verbal consent, participants were given two wristbands to wear for two separate periods of up to 5 days and asked to provide their gender and age. Verbal consent scripts for recruitment of adult and child volunteers are included in the electronic supplementary material. Additionally, we received pesticide use records from 21 participants. Participants in this study were actively working with crops where pesticides are applied. Participants were instructed to seal the wristbands in an individual polytetrafluoroethylene (PTFE) bag with the participant's identification and the dates worn recorded on the bags. Wristbands were returned to the study coordinator (M.S.) and shipped to Oregon State University for analysis. Following analysis, results were communicated back to participants via the study coordinator.

### 2.3. Wristband preparation

Prior to shipment to Diender, Senegal, wristbands were conditioned at 280–300°C for 48 h to remove impurities, then individually packaged in durable, air-tight PTFE bags. Several wristbands from each batch were extracted and analysed by gas chromatography–mass spectrometry before inclusion in the study to ensure they were free of impurities (see *quality control* (QC) below). Following deployment and return to Oregon State University, wristbands were cleaned in sequential baths of 18 MΩ cm^−1^ water and isopropanol to remove superficial fouling or particles. Wristbands were stored in amber glass jars at −20°C for up to one month until extraction. Extraction surrogates were added immediately before extraction, then wristbands were extracted twice in 100 ml ethyl acetate. Extracts were combined and quantitatively reduced to 1 ml.

### 2.4. Chemical analysis

A novel, fast GC-ECD method was developed and validated for analysis of pesticides in passive sampler wristbands. The list of target analytes from a previously described method [9] was expanded and analysis time was shortened, without sacrificing detection limits. Hydrogen was used as carrier gas instead of helium to improve chromatographic resolution. The use of H_2_ reduced analytical cost, increased analytical sensitivity and allowed for decreased analysis time. The current method provides good chromatographic separation in less than 22 min. Further modification of the temperature profile and a 33% reduction of nitrogen make-up gas flow-rate resulted in approximately fivefold increase in sensitivity. In comparison, recently published pesticide GC-ECD methods using helium as the carrier gas [9,16,22], are over 55 min long [9,22,23], or have costlier make-up gases like methane and argon [22]. A less resource-intensive methodology is better adapted to West African laboratories [9].

An internal standard was added to correct for instrument variability, and extracts were analysed using an Agilent 6890N gas chromatograph (GC) with dual 7683 injectors, dual DB-XLB and DB-17MS columns (Agilent, Santa Clara, Cal., USA), and dual micro-electron capture detectors (μ-ECD). Detection limits, quantitation limits and chromatographic conditions are given in the electronic supplementary material, tables S1 and S2. Identification and quantitation were typically made with the DB-17MS column, and the DB-XLB column was used for confirmation. Target compounds were quantified by the relative response of the internal standard to target compound in a 4–6 point calibration curve (*R*^2^ > 0.98, electronic supplementary material, table S1). Two compounds, fenitrothion and malathion were not chromatographically resolved, and thus are reported as a sum. Instrument concentrations were corrected for extraction surrogate recovery and normalized for the mass of the wristband. Examples of standard and wristband extract overspike chromatograms are shown in the electronic supplementary material, figures S1 and S2, illustrating the high degree of separation. Final concentrations are given as nanograms per gram wristband.

### 2.5. Statistical analysis

Compounds with concentrations above detection limit in at least two wristbands were subjected to statistical analysis. For these analyses, any compounds that were below the detection limit were assigned a value equal to one-half of the detection limit. Compounds between detection and quantitation limits were assigned a value of one-half the quantitation limit. Spearman correlation coefficients were computed to evaluate the relationship between individual compounds. One pair was highly correlated with each other (*cis-* and *trans-*permethrin, *ρ* = 0.81; electronic supplementary material, table S3), and these isomers were summed for subsequent analyses. Spearman correlation analysis was also performed to compare concentrations in wristbands worn by each participant in two sequential, 5-day periods. Fisher's exact test and odds ratio analysis was used to compare counts of detected and non-detected, reported and non-reported pesticides. Signed-rank tests were used to compare concentrations and number of detections between wristbands worn by each participant in both periods. Rank-sum tests were used to compare pesticide concentrations in wristbands worn by male and female participants. These non-parametric alternatives to paired and two-sample *t*-tests allow incorporation of left-censored data present in this dataset as values below detection and quantitation limits. Bonferroni adjustments were used to correct for family-wise error rates. Exploratory principal component analysis was also used to evaluate associations between pesticides and selected demographic data to identify risk factors. Statistical analyses were performed with JMP 12.0.1 (SAS Institute, Inc., Cary, NC, USA).

### 2.6. Quality control

To ensure data quality objectives were met, over 40% of samples analysed in this study were QC samples. QC samples included pre-deployed wristband conditioning batch verification (*n* = 2), instrument solvent blanks (*n* = 10), sample matrix overspikes (*n* = 6), sample duplicates (*n* = 2) and continuing calibration verifications (CCVs, *n* = 28). Trip blanks (unopened wristband samples in individual PTFE bags that are shipped to and from the study site alongside the samples) were not returned for analysis. However, in an analogous project in which samples travelled between Oregon and Peru, all wristband trip blank samples were below detection limit for all compounds [24]. Field blanks were not collected in the study. All target pesticide compounds were below detection limit in all blank QC samples. Sample matrix overspikes were within 20% of expected value, and duplicate samples were within 50% relative per cent difference for all detected compounds. Quantitation of CCVs were within data quality objectives of ±30% of true value for 70% of compounds.

## 3. Results

Seventy wristbands were analysed, with 100% compliance among the 35 participants. Thirty participants were male, and five were female. Participants reported the use of 13 pesticide active ingredients during wristband wear, including six that were included in the 63-analyte method (table 1). All six of the pesticides that were both reportedly used and included in the analytical method were detected in at least one wristband. Nineteen pesticide active ingredients were detected beyond those reportedly in use. Participant ages ranged between 15 and 63, averaging 38.

**Table 1.** Counts of pesticide ingredients used by participants. Use data were obtained from 21 of 35 participants, for a total of 42 wristbands. Asterisks indicate compounds that were included in the analytical method.

pesticide active ingredient	times reported (of 42)
dimethoate*	20
acetamiprid*	16
λ-cyhalothrin*	16
imazapyr	14
profenofos	13
*Bacillus thuringiensis*	9
fipronil*	7
dicofol*	5
methomyl	5
methamidophos	3
sulfur	3
azadirachtin	2
deltamethrin*	2

**Figure 1.**
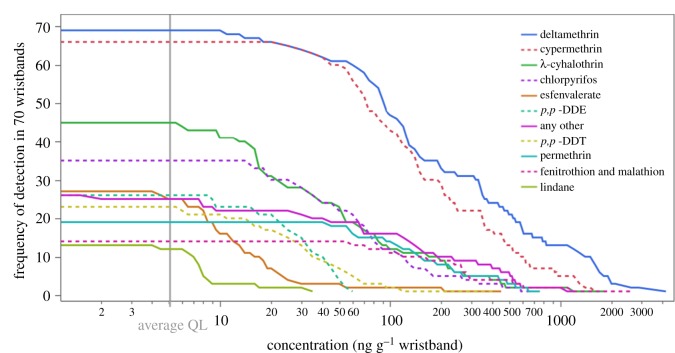
Frequencies of detected pesticides by concentration. Each line represents the frequency that met or exceed a given concentration threshold. Cypermethrin and deltamethrin were above quantitation limit in 69 and 66 of 70 wristbands, respectively. The highest detected concentration was deltamethrin at 4200 ng g^−1^ wristband. Average quantitation limit (QL) for these 10 most frequently detected pesticides, 5.1 ng g^−1^ wristband is highlighted.

Of the 63 pesticide compounds included in the analytical method, 26 were detected in one or more wristbands. Deltamethrin and cypermethrin were the most frequently detected compounds, found in 69 and 66 of 70 wristbands, respectively (figure 1). An analogous figure showing the frequency of detection by participant is included as the electronic supplementary material, figure S3. Each wristband provided between 2 and 10 pesticide detections. Extraction recoveries averaged 66% (range = 11–124%, median = 68%). Such extraction recoveries have been seen previously in silicone wristbands (surrogate PAH recovery 53–122% [20]) and silicone rubber (surrogate pesticide recovery 13–113% [25]). Log octanol–air partition coefficient (log *K*_oa_) of detected pesticides ranged from 5.84 (endosulfan sulfate) to 12.5 (bifenthrin). Log octanol–water partition coefficient (log *K*_ow_) values ranged from 0.78 (dimethoate) to 8.15 (bifenthrin) (electronic supplementary material, table S1) [26]. Of the 383 total pesticide detections in the 70 wristbands, 97% were insecticide or insecticide degradation products or metabolites, and 63% of the insecticide detections were synthetic pyrethroids. The remaining 3% of total detections were herbicide and fungicide active ingredients.

**Figure 2.**
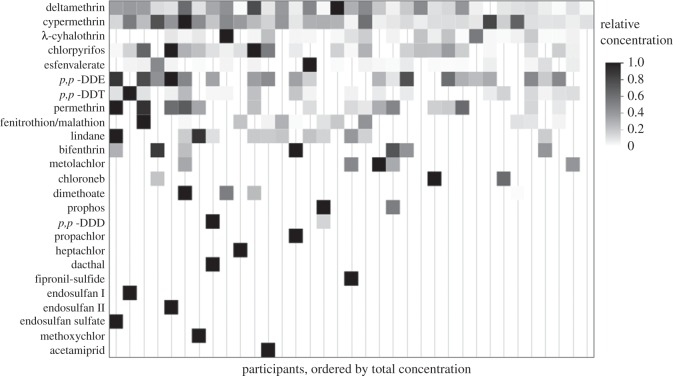
Concentrations of the detected pesticides, each on a relative scale. Boxes from palest to darkest indicate the concentration range of pesticides detected above quantitation limit. Pesticide concentrations in wristbands worn in two periods are averaged for each participant. Participant order was arbitrarily assigned, and gender is not given in order to maintain participant anonymity.

Detection of a pesticide sequestered within a silicone wristband represents an individualized, composite dermal and inhalation exposure that is suitable for comparison among participants. Relative concentrations from below detection limit to maximum amount are depicted as a heat map (figure 2). Comparisons of concentrations *within* one wristband should be made with caution, as greater concentrations of different pesticides may be the result of several factors beyond personal exposure, including uptake kinetics (i.e. sampling rate) and partition coefficients (e.g. log *K*_ow_), which have been shown to be inversely correlated [27]. For example, we expect dimethoate to have a higher sampling rate than bifenthrin, based on their relative *K*_ow_ values. Specific sampling rates are not determined in this study, so comparing bifenthrin concentrations to dimethoate concentrations is inappropriate. Concentrations on an absolute scale are depicted in the electronic supplementary material, figure S4. Conversely, the uptake rate of bifenthrin will be approximately equivalent for all participants, and comparisons of bifenthrin between study participants is reasonable. The highest concentration of any pesticide measured was deltamethrin at 4200 ng g^−1^ wristband (figure 1).

**Figure 3.**
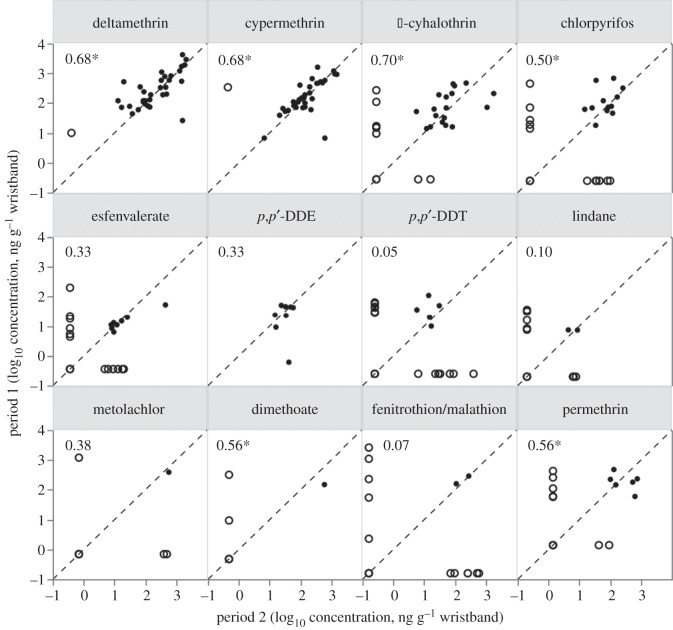
Comparison of concentrations in wristbands worn by participants in two sequential periods of up to 5 days. Dashed line represents 1 : 1 relationship, and open circles indicate when pesticide was detected in only one wristband. Spearman correlation coefficients are given, where asterisks indicate significant *p*-values after Bonferroni adjustment <0.003. Data are not shown if below limit of detection in both wristbands.

Each participant wore a wristband for two separate periods. Wristbands were worn for up to 5 days in each period, with 94% worn for either 4 or 5 days. Concentrations were not adjusted for the duration of wear because the vast majority were worn for a similar length of time. Neither the number of positive detections nor the concentrations of individual pesticides sequestered in a participant's wristband were different between the two periods (signed-rank test, no significant *p*-values after Bonferroni adjustment less than 0.003). Concentrations between the two periods were correlated for five pesticides: deltamethrin, cypermethrin, *λ*-cyhalothrin, chlorpyrifos and dimethoate (figure 3, Spearman's rho correlation, significant *p*-value after Bonferroni adjustment less than 0.003). Pesticides with fewer overall detections were less strongly correlated. This analysis yielded similar results when repeated omitting data below detection limit (electronic supplementary material, table S4).

For the 42 wristbands worn by the participants with pesticide use data, there were a total of 2016 possible detections in the analytical method: 42 samples × 48 analytes (after combining isomers, degradation products and metabolites). Counts of reported and non-reported, and detected and non-detected data are summarized in table 2. The odds of a pesticide being detected are 4.3 times greater if it was reportedly used (95% CI: 2.5–7.3; Fisher's exact two-tailed *p*-value < 0.001). Quantitative analysis comparing the magnitude of concentrations against use reports was not possible because of little overlap of pesticides both detected and reported. Approximately 10% of positive detections were also reported to be in use by participants during wristband wear. Nineteen pesticide active ingredients were detected beyond those reportedly in use.

**Table 2.** Contingency table of counts and total percentages for the 42 wristband samples worn by 21 participants with pesticide use reports. Total sum is 2016; 42 samples × 48 analytes, after combining isomers, degradation products and metabolites.

	reported	not reported
detected	21 (1%)	192 (10%)
not detected	45 (2%)	1758 (87%)

Rank-sum tests were used to compare pesticide concentrations in wristbands worn by male (*n* = 60) and female (*n* = 10) participants. Two pesticides showed significant *p*-values after Bonferroni adjustment (*p* < 0.003, electronic supplementary material, figure S5). Deltamethrin and λ-cyhalothrin had higher concentrations in male participants' wristbands than females. Identical significance conclusions are obtained when this analysis is repeated with concentrations that have been normalized to (divided by) the number of days the wristbands were worn.

Exploratory principal component analysis (PCA) results did not reveal any strong demographic groupings among the detected pesticides (electronic supplementary material, figure S6). Wristbands worn by female participants are clustered slightly; however, principal components 1 and 2 cumulatively explained only 22.1% of the variance. No other clusters were present that would represent either participant age or the number of days that wristbands were worn (data not shown).

## 4. Discussion

The majority of analytes in the instrument method were insecticides because they were expected to be used most frequently. The 63-analyte method included 38 insecticides, 10 herbicides, 7 fungicides and 8 other chemicals, e.g. degradation products. Williamson *et al*. [5] determined that insecticides made up 55% of reported active ingredients through surveys in Senegal and three other African countries. They suggest this is attributable both to the greater severity of insects compared with other pests, and that insecticides are more available and less expensive than other pesticides. The bias towards insecticides in this study aligns with the expectation that pesticides targeting insect pests are the most commonly used. Furthermore, the wristband and analytical methods of this study detected seven of the top nine active ingredients in Williamson *et al.* [5], suggesting that the methods are effective at determining exposure to relevant pesticides. Williamson *et al*. [5] also found that 33–60% of villagers in a cowpea- and cotton-farming village in Ghana reported ill health effects each season associated with exposure to endosulfan, chlorpyifos and λ-cyhalothrin. Though the geographical location described differs from this study, all three of these pesticides were detected in one or more wristband. The wristbands sequestered the pesticides that are most likely to have effects on human health. Ideally, the 63-pesticide method would include all pesticides sold in the West African market, e.g. methamidophos which is believed to be a major driver of risk in similar communities [6], but was not included in the method because it was not amenable to detection via GC-ECD.

We detected in at least one wristband, all six of the pesticides that were both reportedly used and included in the analytical method. The most frequently reported pesticide, dimethoate is the least hydrophobic analyte in the method. In comparison with the more hydrophobic analytes, it is expected that a higher level of exposure is required for appreciable accumulation in the wristband polymer. Dimethoate was detected in only four wristbands despite being used by 15 of 21 reporting participants. Similarly, acetamiprid was frequently used, but only detected above quantitation limit in one wristband. The electron capture detector is less sensitive to acetamiprid, whose quantitation limit is more than 10 times higher than the average limit for the other compounds. For these two compounds, exposures are likely to have occurred without detection. Both of these examples highlight that the passive sampling wristband polymer as well as the chosen analytical method play a role in the resulting exposure profiles in this study.

Beyond those reported by participants for use in agriculture, an additional 19 pesticide active ingredients were detected, notably cypermethrin and chlorpyrifos. These and other pesticides may not have been reported because they are not used in food crops, but rather for pest control within the home, on domestic animals, or in forage crops intended for animal consumption. Additional exposures may be the result of improper pesticide storage, contaminated equipment or unknown use in neighbours' crops. Finally, the farmers may not always know what they have applied because of illiteracy or improper/missing labels ([6,28], M.L.H. 2016, personal observation).

Overall, variation in exposure profiles was highly individualized with only small effects that suggest participant gender may be a risk factor for increased exposure. Only one demographic trend was identified in which two pesticides, deltamethrin and *λ*-cyhalothrin, were detected in greater amounts in wristbands worn by men; however, small sample size and unbalanced gender distribution may limit inferences. The number of pesticide detections as a function of number of days worn was also greater among male participants. A focus group with six women (ages 23–40) was conducted in March 2015 within the study area to gain understanding of the role women play in farming and pesticide management. This revealed that some women routinely apply pesticides on the part of the family farm they manage (M.L.H. 2016, personal communication). This information suggests both genders may have the same chance for exposure, but actual exposure can vary by the identity of crops managed by men and women.

The wristband technology allowed us to detect highly individualized exposure profiles for the participants in the study. Neither the number of pesticides detected nor the concentrations differed between the two sampling periods. Periods of wear were chosen at the discretion of the participants; however, all had completed the first period by the midpoint of the study on 25 November 2014. With no temporal overlap between the first and second periods by any participants, these findings reveal that no distinct trend in pesticide profiles occurred before or after the midpoint of the study. Exposure profiles for both periods were therefore averaged for each participant, and the individualized results depicted in figure 2 corroborate the finding that the presence of one pesticide does not correlate with the presence of another. Results from both signed-rank and Spearman's correlation analyses (figure 3) reveal two wristbands worn by any one individual yield similar results. Additionally, the limited results of PCA highlight the highly individualized profiles among participants. Despite being worn by the same individuals, the paired wristband samples are not true replicates because the dates worn do not coincide. Regardless, multiple analyses reveal that the greatest variability in exposure profiles is among individuals, not between wristbands worn by the same individual.

**Figure 4.**
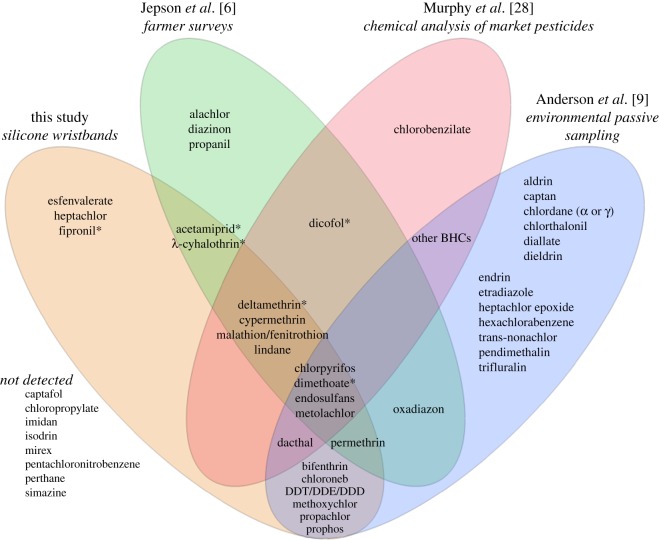
Comparison of pesticides detected in this study to previous pesticide use studies in West Africa, with survey data of village farmers, 2007 and 2010 [6]; analysis of vector control pesticides sold in markets in the Gambia, 2005 [28] and environmental water sampling with passive samplers, 2012 [9]. Only pesticides included in the 63-pesticide method are included here, and lists of further pesticide detections in comparable studies are given in the electronic supplementary material. Asterisks indicate compounds that were reported in use by participants as listed in table 1.

Pesticide detections in the present investigation could be compared with three other studies (figure 4): Jepson *et al.* [6] administered surveys to village farmers in five West Africa countries in 2007 and 2010; Murphy *et al.* [28] analysed vector control pesticides collected from markets in the Gambia in 2005; and Anderson *et al.* [9] deployed passive samplers in water near agriculture in five West Africa countries. Isomers, degradation products and metabolites are combined to better enable comparisons. Only the 63 pesticides in the described analytical method are included in figure 4; a list of compounds found in those studies but not included in the present analysis can be found in the electronic supplementary material. Three pesticides were detected only in this study and not reported in the three comparator studies: esfenvalerate, heptachlor and fipronil. Esfenvalerate was detected in 27 wristbands, and although it was not reported to be used in this study or recent surveys [6], it is known to be available in the region (M.S. 2016, personal observation). The presence of heptachlor in a single wristband may be attributable to its environmental persistence, supported by a recent report of heptachlor detections in Senegal estuary sediments [29]. Fipronil was also detected in a single wristband and has been used to control locusts, grasshoppers and animal parasites in this region (P.C.J. 2016, personal observation).

Surveys administered by Jepson *et al.* provide details of regional pesticide use practices. Compounds that were detected in wristbands but absent in Jepson *et al*. [6] highlight legacy contaminants or pesticides that farmers are unaware are being used, e.g. esfenvalerate, bifenthrin and DDT and its breakdown products. We also include a comparison with Murphy *et al.* [28] as the best available analysis of pesticides in the regional markets. Active ingredients identified in [28] are intended for vector control, rather than a focus on agricultural pesticides as in this study. However, the vector control pesticides were purchased in an informal market, and therefore represent what may be available for purchase by village farmers. Murphy *et al.* [28] were unlikely to be hampered by analytical detection limits because they were analysing a packaged product, whereas compounds sequestered in the wristband have been subjected to varying degrees of dilution and degradation. Finally, a small percentage of collected samples were not identifiable in the GC/MS analysis used by Murphy *et al.* [28].

The analytical method used is an expansion of that used in Anderson *et al.* [9]. In addition to an extended list of analytes, further differences in detected compounds between Anderson *et al.* [9] and this work may be a result of several factors: duration of deployment (less than or equal to 5 days versus 14 days), dates of deployment (2014 versus 2012), identity of the sampled matrix (air/dermal versus water), material of the passive sampler (silicone versus low-density polyethylene), different study location (Senegal alone, versus greater West Africa including Senegal), and different analytical parameters. Nevertheless, almost half of the pesticides detected in wristbands were also found using stationary environmental passive sampling. The three most frequently detected pesticides, deltamethrin, cypermethrin and *λ*-cyhalothrin, were not included in chemical analysis methods of Anderson *et al.* [9].

Certain pesticide active ingredients with increased potential for human or environmental harm are subject to international negotiations and restriction. Countries or parties adhering to the Rotterdam Convention may only export a listed chemical with the prior informed consent of the importing country or party [30,31]. The Stockholm Convention compels countries to phase out the production, trade and use of several chlorinated persistent organic pollutants [31]. The wristbands sequestered three pesticides that are subject to both the Rotterdam and Stockholm conventions: DDT, heptachlor and lindane (electronic supplementary material, table S5). Senegal, under the Rotterdam Convention, does not consent to allow imports of DDT or heptachlor but does consent lindane to be imported conditionally [32]. Under the Stockholm Convention, lindane use is permissible for human pharmaceutical treatment of head lice and scabies [33]. The detection of lindane in wristbands worn in Senegal is not therefore unexpected because the use is permitted, although environmental persistence from past applications may also explain our detections. No exemptions in Senegal are permitted for heptachlor under either convention, but it is a legacy contaminant that has been detected as recently as 2008 in environmental samples in Senegal [29]. It is likely that the single detection of heptachlor in a wristband is the result of historic use.

DDT was not found in survey data from West African farmers [6], or in analysis of vector control pesticides available in adjacent Gambian markets [28], and it is our understanding that DDT is no longer used in Senegal (M.S. 2016, personal observation). Under the Stockholm Convention, DDT may be used in the event of a plague [33], and an estimated 0.3 tonnes of DDT are stockpiled in the event of malaria outbreaks [34]. Whether or not DDT has been used in recent decades, the presence of DDT and its degradation products was expected because of its environmental persistence. Diagnostic ratios such as DDT/(DDE + DDD) are frequently calculated in environmental sampling campaigns in order to estimate the relative age of DDT applications, where values more than 1 suggest more recent DDT applications [35–37]. In this dataset, comparisons of concentrations within one wristband sample must be made with caution because pesticide compounds have different sampling rates that cannot be estimated without the use of *in situ* calibration standards (performance reference compounds, PRCs). Relative sampling rates were approximated from previous research that found the sampling rate of DDT by passive sampling devices to be 10–20% less than that of DDE or DDD [10,38]. When the above ratio is calculated for the 36 wristbands that contained one or more of DDT, DDE or DDD, 16 had a ratio that suggests recent DDT application. This count increases to 17 when the DDT concentration is mathematically increased by 20%. Both the ratios of the absolute values and the adjusted values give an evenly mixed distribution of old and new signatures.

The analytical method included nine ‘active ingredients believed to be obsolete or discontinued for use as pesticides’ by the World Health Organization [30] (electronic supplementary material, table S5). Of these, two pesticides chloroneb and heptachlor were detected in 4 and 1 wristbands, respectively. Neither was reportedly used, but the presence of heptachlor may be expected due to its long environmental half-life [29]. In total, legacy and obsolete pesticides (DDT, heptachlor, lindane and chloroneb) compromised 18% of total detections among all wristband samples.

Three pesticides included in instrumental analysis are classified by the WHO as Class Ia (extremely hazardous); however, none were detected in any samples: captafol, prophos (ethoprophos) and hexachlorobenzene. The majority of target analyte pesticides are Class II (moderately hazardous), while others were Class III (slightly hazardous) (electronic supplementary material, table S1). Of the reportedly used pesticides (table 1), methamidophos and methomyl are Class Ib (highly hazardous), while the remainder are either Class II or Class III.

Pyrethroid insecticides compromised the majority of detections in wristband samples. These compounds are widely used because they have low toxicity to humans but are highly toxic to insects. Common uses include insect repellents in clothes and mosquito nets, topical treatment for head lice, and agricultural use as replacement for some organophosphate (OP) pesticides. Type II pyrethroids (e.g. cypermethrin, deltamethrin, *λ*-cyhalothrin and esfenvalerate) are generally more toxic to insects and mammals than Type I (e.g. bifenthrin and permethrin) [39–41]. The silicone wristbands incorporate both inhalation and dermal exposures by sequestering compounds in air and in direct contact with skin or materials/solutions that touched the skin that contain lipophilic compounds. Inhalation and dermal exposure to pyrethroids are linked to respiratory, neurological and skin effects [39–41], while more recent reports investigate male reproductive effects or exposures during pregnancy [41]. The high frequency of pyrethroid pesticides detected among participants clearly indicates a pervasive presence in the participants' immediate environment.

OP pesticides, predominantly chlorpyrifos, were detected in over half of the wristband samples. Higher levels of urinary OP metabolites in pregnant mothers have been linked to impaired cognitive development [42,43] and remain the subject of ongoing epidemiological research [44]. Frequently, OP pesticide exposure is estimated in epidemiological studies by urinary dialkyl phosphate concentrations. The use of these metabolites as biomarkers has limitations, in particular, that dialkyl phosphates are not specific to individual OP pesticide compounds from which they are derived [45]. Furthermore, dialkyl phosphates can be formed directly on food products, and cannot be differentiated from those formed during metabolism [44]. Parent OP pesticide compounds can be identified using the silicone wristband, and they represent opportunity for both dermal and inhalation exposure.

The ease-of-use of the wristbands by the participants resulted in 100% compliance. Participant training was accomplished quickly, and the wristbands were worn for days, indicating a relative ease of compliance and incorporating agricultural, domestic and other exposure sources. Ease-of-use of passive samplers has been described previously [46,47], although not at the personal level. Easy training and high compliance rates suggest that the silicone wristbands represent a promising tool for establishing baseline data in pesticide risk management education, a necessary, yet often missing piece of information when evaluating, for example, the effectiveness of farmer field schools [48]. They may also be used to verify risk management decisions that farmers make following education, and also determine the degree to which farmers and their families can reduce pesticide exposure through their own decisions [8].

A further benefit of this method is ease of sample transportation to and from the study location. O'Connell *et al.* [20] demonstrated the stability of five model compounds sequestered in wristbands under simulated transport conditions in PTFE bags at 35°C for up to 72 h, mimicking conditions of overseas shipment, and also representing common conditions of transport in West Africa [20]. In comparison, hand-wash and patch samples are transported and stored in glass containers under refrigeration, or on ice [16]. Biological samples including blood and urine also need to be transported quickly on ice [49]. The lightweight, easily transportable wristband passive samplers are better-suited for human exposure assessments in remote locations where expedient transport on ice is costly or even impossible.

The chosen analytical method and silicone wristband material have limitations that influence which pesticides can be detected. Chemicals measured by gas chromatography must have thermal stability and be at least partially volatilized at a nominal 300–350°C. The selected detector further constrains analysis. In this study, ECDs were used because they have greater sensitivity to electronegative elements and functional groups that are common in many organic pesticides [50], and simultaneous analysis with confirmation on two columns minimizes the possibility of false positives. The silicone material of the wristband is intended for sequestration of lipophilic compounds, though it may sequester compounds with lower *K*_ow_ values through splashing or direct contact with skin. For instance, caffeine (log *K*_ow_ = −0.07), which is not very lipophilic, was detected in the initial demonstration of the silicone wristband as a personal passive sampler [20]. In this study, the detected compound with the lowest log *K*_ow_ was dimethoate (log *K*_ow_ = 0.78), found in a single wristband.

This is the first direct measurement of individualized pesticide exposure in West Africa, but there are limitations to consider when drawing inferences. First, the limited sample size may not be representative of a wider population, as participants were recruited as volunteers and do not represent a random sample. Statistical power in comparisons between genders is limited because male participants outnumbered female six to one. Analysis of a trip blank was not included in this study, but we expect blank results as measured in a similar study [24]. The wristband is a proxy for exposure that incorporates both dermal and vapour-phase inhalation routes, and it does not consider oral and dietary pathways of exposure. Additionally, wristbands were cleaned to remove particles that adhered to the wristband surface. This excludes pesticides that may be toxicologically relevant when inhaled while adsorbed to airborne particles. It is intrinsically difficult to interpret what portion of the pesticide load in a wristband aligns with different routes of exposure. Performance reference compounds (PRCs, or depuration compounds) are often used as *in situ* calibration standards in environmental passive sampling campaigns. PRCs are infused into the passive sampler before deployment, and the rate at which PRCs diffuse out is correlated to the rate at which environmental compounds are sequestered [1]. Because the wristbands sample multiple media, PRC loss, and thus compound uptake, cannot be linked definitively to either air for an inhalation exposure or to skin for a dermal exposure. Finally, chemical uptake into a wristband is anticipated to be affected by differing environmental conditions among participants that were not measured in this study, e.g. temperature and wind/air speed. However, the hydrophobic silicone material of the wristband mimics biological membranes, and factors that increase uptake into the wristband should coincide with increased exposure for participants.

## 5. Conclusion

Silicone wristbands sampled personal exposure to a broad range of agricultural, domestic, legacy and current-use pesticides and provided the first report of individualized exposure profiles among smallholder farmers in West Africa. Reports of personal pesticide exposure in West Africa have been lacking because of difficulties in sampling logistics, e.g. participant compliance with invasive methods, the difficulties of sample shipment and the lack of analytical capacity in the region. Every wristband sequestered two or more pesticides, demonstrating both the individualized nature of pesticide exposure in the sampled population, and the sensitivity of the wristbands and analytical method. An additional 19 pesticide compounds were detected beyond those that were reported to be in use, highlighting the importance of measuring exposure in addition to collecting surveys and self-reported use records. Future surveillance systems for pesticide exposure and health effects in West Africa will require reliable and standardized methods that are within the capacity of local institutions and which support decision makers including regulators, policy makers, educators and medical practitioners [21]. The methodology that we outline here represents the first practical solution to these challenges in West Africa.

## Supplementary Material

SI_1.docx contains: pesticide identities and physiochemical properties, chromatographic conditions, Spearman's rho correlation coefficients, a list of analyzed pesticides subject to international treaties, example chromatograms, individualized pesticide concentrations, concentration distributions by gender, results of principal component analysis, and verbal consent scripts.

## Supplementary Material

SI_2.csv contains the dataset. Concentrations are ng/g wristband. Compounds below the detection limit were assigned a value equal to 1/2 detection limit. Compounds between detection and quantitation limits were assigned a value of 1/2 quantitation limit. Gender and age data are removed to maintain participant anonymity.

## Data accessibility

The dataset supporting this article has been uploaded as the electronic supplementary material. Gender and age data are not included to maintain anonymity. The following information is also available in the electronic supplementary material: pesticide identities and physiochemical properties, chromatographic conditions, Spearman's rho correlation coefficients, a list of analysed pesticides subject to international treaties, example chromatograms, individualized pesticide concentrations, concentration distributions by gender, results of principal component analysis and verbal consent scripts.
