# Supplementary material for: Silicone wristbands detect individuals' pesticide exposures in West Africa
Source: R Soc Open Sci. 2016 Aug 17;3(8):160433. doi: 10.1098/rsos.160433 (PMC5108971; doi:10.1098/rsos.160433)
Supplement: SI_1.docx contains: pesticide identities and physiochemical properties, chromatographic conditions, Spearman's rho correlation coefficients, a list of analyzed pesticides subject to international treaties, example chromatograms, individualized pesticide concentrations, concentration distributions by [file rsos160433supp1.docx]

Silicone wristbands detect an individual’s pesticide exposures in Africa

*Carey E. Donald^a^, Richard P. Scott^a^, Kathy L. Blaustein^b^, Mary L. Halbleib^b^, Makhfousse Sarr^c^, Paul C. Jepson^b^, and Kim A. Anderson^a^**

Supplementary Information

**Note**: *See SI_2.csv for dataset.* Concentrations are ng/g wristband. Compounds below the detection limit were assigned a value equal to 1/2 detection limit. Compounds between detection and quantitation limits were assigned a value of 1/2 quantitation limit. Gender and age data are removed to maintain participant anonymity.

**Table of Contents.**

*Pages 2-3.* **SI Table S1.** Pesticide identities and physiochemical properties.

*Page 4.* **SI Table S2.** Chromatographic conditions.

*Page 5.* **SI Table S3.** Spearman’s rho coefficients.

*Page 6.* **SI Table S4.** Active ingredients included in the 63-analyte GC-ECD method that are believed to be obsolete or discontinued for use as pesticides, or subject to the Rotterdam or Stockholm Conventions [1].

*Page 7*  **SI Figure S1.** Example standard chromatogram.

*Page 8.* **SI Figure S2.** Example wristband overspike chromatogram.

*Page 9.* **SI Figure S3.** Average concentration of detected pesticides by participant.

*Page 10.* **SI Figure S4.** Principle component analysis.

*Page 11.*  Additional pesticides detected in comparison studies.

*Page 12.*  **References**

**SI Table S1.** Pesticide identities and physiochemical properties. Compounds were quantified against a 6-point calibration curve unless otherwise noted.

| Class | Pesticide compound | CAS | DL^e^  (ng/g WB) | QL^f^  (ng/g WB) |  | log K_oa_ [2] |  | log K_ow_  [2] |  | MW (Da) | WHO  Class^g^ [1] |
| --- | --- | --- | --- | --- | --- | --- | --- | --- | --- | --- | --- |
| Insecticides |  |  |  |  |  |  |  |  |  |  |  |
|  | acetamiprid | 135410-20-7 | 49 | 730 | ^b^ | 8.10 | ^c^ | 2.55 | ^c^ | 222.7 | ^d^ |
|  | aldrin | 206-215-8 | 0.75 | 11 |  | 8.08 |  | 6.50 |  | 364.9 | O |
|  | *α*-BHC | 119911-70-5 | 0.046 | 0.69 |  | 8.84 |  | 3.72 |  | 290.8 | II |
|  | *β*-BHC | 319-85-7 | 0.19 | 2.9 |  | 8.84 |  | 3.80 |  | 290.8 | II |
|  | *δ*-BHC | 319-86-8 | 0.48 | 7.2 |  | 8.84 |  | 3.78 |  | 290.8 | II |
|  | bifenthrin | 82657-04-3 | 0.84 | 13 |  | 12.54 | ^c^ | 8.15 | ^c^ | 422.9 | II |
|  | chlorobenzilate | 510-15-6 | 2.1 | 32 |  | 10.27 | ^c^ | 4.72 |  | 325.2 | O |
|  | *α* -chlordane | 5103-71-9 | 0.10 | 1.5 |  | 8.92 |  | 6.16 |  | 409.8 | II |
|  | *γ*-chlordane | 5566-34-7 | 0.20 | 3.0 |  | 8.92 |  | 6.22 |  | 409.8 | II |
|  | chloropropylate | 1437871 | 3.3 | 49 |  | 10.89 | ^c^ | 4.41 | ^c^ | 339.2 | O |
|  | chlorpyrifos | 2921-88-2 | 0.51 | 7.6 |  | 8.88 | ^c^ | 4.96 |  | 250.6 | II |
|  | *λ*-cyhalothrin | 91465-08-6 | 0.55 | 8.3 |  | 11.22 | ^c^ | 6.80 |  | 449.9 | II |
|  | cypermethrin | 52315-07-8 | 0.89 | 13 |  | 10.83 | ^c^ | 6.60 |  | 416.3 | II |
|  | *p,p'*-DDT | 50-29-3 | 0.50 | 7.4 |  | 9.82 |  | 6.91 |  | 354.5 | II |
|  | deltamethrin | 52918-63-5 | 0.81 | 12 |  | 9.90 | ^c^ | 6.20 |  | 505.2 | II |
|  | diazinon | 333-41-5 | 3.5 | 52 |  | 9.15 | ^c^ | 3.81 |  | 304.3 | II |
|  | *o,p'*-dicofol | 10606-76-9 | 0.64 | 9.6 |  | 13.45 | ^c^ | 5.81 | ^c^ | 370.5 | II |
|  | *p,p'*-dicofol | 115-32-2 | 2.6 | 39 |  | 10.03 | ^c^ | 5.02 |  | 370.5 | II |
|  | dieldrin | 60.57-1 | 0.16 | 2.3 |  | 8.13 |  | 5.40 |  | 380.9 | O |
|  | dimethoate | 60-51-5 | 0.94 | 14 |  | 9.15 | ^c^ | 0.78 |  | 229.3 | II |
|  | endosulfan I | 959-98-9 | 0.077 | 1.2 |  |  | ^d^ | 3.59 | ^c^ | 406.9 | II |
|  | endosulfan II | 33213-65-9 | 0.18 | 2.7 |  | 8.64 |  | 3.83 |  | 406.9 | II |
|  | endrin | 72-20-8 | 0.34 | 5.0 |  | 8.13 |  | 5.40 |  | 380.9 | O |
|  | esfenvalerate | 66230-04-4 | 0.72 | 11 |  | 10.97 | ^c^ | 6.20 |  | 419.9 | II |
|  | fipronil | 120068-37-3 | 1.2 | 19 |  | 11.46 | ^c^ | 4.00 |  | 437.1 | II |
|  | heptachlor | 76-44-8 | 0.71 | 11 |  | 7.64 |  | 5.47 |  | 373.3 | O |
|  | imidan (phosmet) | 732-11-6 | 0.24 | 3.6 |  | 9.25 | ^c^ | 2.78 |  | 317.3 | II |
|  | isodrin | 370-14-9 | 0.14 | 2.1 |  | 9.74 | ^c^ | 1.74 | ^c^ | 165.2 | O |
|  | lindane (*γ*-BHC) | 58-89-9 | 0.40 | 6.0 |  | 8.84 |  | 4.14 |  | 290.8 | II |
|  | malathion/  fenitrothion | 121-75-5/  122-14-5 | 0.31 | 4.7 |  | 9.06/  7.72 | ^c^ | 2.63/  3.30 |  | 330.4/  277.2 | III/  II |
|  | methoxychlor | 72-43-5 | 0.70 | 11 |  | 10.16 | ^c^ | 5.08 |  | 345.6 | U |
|  | mirex | 2385-85-5 | 0.51 | 7.6 |  | 8.37 | ^c^ | 6.89 |  | 545.5 | O |
|  | *trans*-nonachlor | 39765-80-5 | 0.056 | 0.84 |  | 9.66 |  | 6.35 |  | 444.2 | ^d^ |
|  | *cis*-permethrin | 61949-76-6 | 1.7 | 26 |  | 10.17 | ^c^ | 6.50 |  | 391.3 | II |
|  | *trans*-permethrin | 51877-74-8 | 1.1 | 16 |  | 10.62 | ^c^ | 6.50 |  | 391.3 | II |
|  | perthane (perthan) | 72-56-0 | 11 | 161 | ^a^ | 8.19 | ^c^ | 6.66 | ^c^ | 307.3 | ^d^ |
|  | prophos (ethoprophos) | 13194-48-4 | 2.3 | 35 |  | 8.77 | ^c^ | 3.59 |  | 242.3 | Ia |
| Herbicides |  |  |  |  |  |  |  |  |  |  |  |
|  | alachlor | 15972-60-8 | 0.60 | 9.0 |  | 9.99 | ^c^ | 3.52 |  | 269.8 | II |
|  | dacthal (chlorthal-dimethyl) | 1861-32-1 | 0.38 | 5.7 |  | 8.33 | ^c^ | 4.28 |  | 332.0 | III |
|  | diallate | 2303-16-4 | 2.2 | 33 | ^a^ | 8.23 | ^c^ | 4.49 |  | 270.2 | O |
|  | metolachlor | 51218-45-2 | 1.4 | 21 | ^a^ | 9.33 | ^c^ | 3.13 |  | 283.8 | III |
|  | oxadiazon | 19666-30-9 | 5.0 | 75 |  | 10.33 | ^c^ | 4.80 |  | 345.2 | U |
|  | pendimethalin | 40487-42-1 | 1.4 | 20 | ^a^ | 18.84 | ^c^ | 2.62 | ^c^ | 281.3 | II |
|  | propachlor | 1918-16-7 | 2.4 | 37 |  | 7.61 | ^c^ | 2.18 |  | 211.7 | II |
|  | propanil | 709-98-8 | 0.70 | 10 |  | 9.23 | ^c^ | 3.07 |  | 218.1 | II |
|  | simazine | 122-34-9 | 4.4 | 66 |  | 9.59 | ^c^ | 2.18 |  | 201.7 | U |
|  | trifluralin | 1582-09-8 | 0.63 | 9.4 |  | 7.72 | ^c^ | 5.34 |  | 335.3 | U |
| Fungicides |  |  |  |  |  |  |  |  |  |  |  |
|  | captafol | 2425-06-1 | 49 | 730 | ^b^ | 10.87 | ^c^ | 3.80 |  | 349.1 | Ia |
|  | captan | 133-06-2 | 0.26 | 3.9 |  | 9.34 | ^c^ | 2.80 |  | 300.6 | U |
|  | chloroneb | 2675-77-6 | 2.2 | 33 |  | 6.81 | ^c^ | 3.44 | ^c^ | 207.1 | O |
|  | chlorothalonil | 1897-45-6 | 0.43 | 6.5 |  | 7.14 | ^c^ | 3.05 |  | 265.9 | U |
|  | etridiazole | 2593-15-9 | 0.36 | 5.4 |  | 8.31 | ^c^ | 3.37 |  | 247.5 | III |
|  | hexachlorobenzene | 118-74-1 | 0.23 | 3.4 |  | 7.38 |  | 5.73 |  | 284.8 | Ia |
|  | pentachloronitrobenzene (quintozene) | 82-68-8 | 0.74 | 11 |  | 7.38 | ^c^ | 4.64 |  | 295.3 | U |
| By-products |  |  |  |  |  |  |  |  |  |  |  |
|  | endosulfan sulfate | 1031-07-8 | 0.22 | 3.2 |  | 5.84 | ^c^ | 3.66 |  | 422.9 | ^d^ |
|  | endrin aldehyde | 7421-93-4 | 0.45 | 6.7 |  | 11.20 | ^c^ | 5.73 | ^c^ | 382.9 | ^d^ |
|  | endrin ketone | 53494-70-5 | 2.3 | 35 |  | 11.07 | ^c^ | 4.99 | ^c^ | 380.9 | ^d^ |
|  | heptachlor epoxide | 1024-57-3 | 0.44 | 6.7 |  | 8.05 | ^c^ | 4.98 |  | 389.3 | ^d^ |
|  | *p,p'*-DDD | 72-54-8 | 0.27 | 4.0 |  | 10.10 |  | 6.02 |  | 320.0 | ^d^ |
|  | *p,p'*-DDE | 72-55-9 | 0.081 | 1.2 |  | 9.86 |  | 6.51 |  | 318.0 | ^d^ |
| Metabolites |  |  |  |  |  |  |  |  |  |  |  |
|  | fipronil sulfide | 120067-83-6 | 0.75 | 11 |  | 16.51 | ^c^ | 4.82 | ^c^ | 421.1 | ^d^ |
|  | fipronil sulfone | 120068-36-2 | 0.80 | 12 |  | 18.21 | ^c^ | 4.42 | ^c^ | 453.1 | ^d^ |

*^a^ 5-point calibration*

*^b^ 4-point calibration*

*^c^ estimate*

*^d^ data not available*

*^e^ Detection limit, determined as described in SI Table S2*

*^f^ Quantitation limit, determined as described in SI Table S2*

*^g^* U: *unlikely to present acute hazard in normal use.* O: *believed to be obsolete or discontinued for use as a pesticide.* Ia, II, *and* III: *defined as in [1].*

**SI Table S2**. Chromatographic conditions and methodology.

|  |  |
| --- | --- |
| Injectors | Each 10 μL injector needle was washed with three alternating hexane and acetone aliquots, before and after each sample in order to remove sample carry-over. Injector needles were both set for fast plunger speed. |
| Inlets | Inlet temperature set at 250°C, and inlets were equipped with 4mm ID liners with a single taper. Inlets were purged after injection at 40 mL/min for 0.75 min. |
| Carrier gas | Hydrogen carrier gas was used, with a linear flow rate of 72 mL/min. |
| Columns | Both Agilent DB-XLB and Agilent DB-17MS capillary columns were 30 m in length, 0.25 mm diameter, and a 0.25μm film thickness. |
| Oven | Hold at 110°C for 0.5 minute, ramp to 150°C at 25°C/min, ramp to 229°C at 6°C/min, ramp to 320°C at 20°C/min, and hold at 320°C for 2.5 minutes. |
| Detectors | μ-ECDs set to 320 °C with combined column flow and detector make-up gas set to 40 mL/min, where make-up gas was 99.999% nitrogen. |
| Software | Data analysis was performed using Agilent Chemstation version E.02.00.493. |
| Confirmation process | The process of confirmation includes identifying the target analyte by comparing the retention time of the peak on both the DB-17MS chromatogram with those peaks in the standard and sample on the second DB-XLB column chromatogram. A confirmed target analyte that has a value above the quantitation level and comparable area (and shape) on both chromatographic columns and appropriate retention times was considered confirmed. By comparing these additional data elements, peak size, shape and retention time for both columns, false identification can be reduced. |
| Determination of detection and quantitation limits | A low standard, 10 ug/L, was analyzed a minimum of 7 times. Detection limits were calculated as 3 X standard deviation. Quantitation limits were then determined to be 15 X detection limit, reported in SI Table S1. |

**SI Table S3.** Spearman’s rho coefficients among concentrations of pesticides detected above quantitation limits in at least 2 wristbands. Bold-face values are significant (p < 0.05).

|  | cypermethrin | λ-cyhalothrin | chlorpyrifos | esfenvalerate | p,p'-DDE | p,p'-DDT | trans-permethrin | malathion/  fenitrothion | lindane (γ-BHC) | cis-permethrin | bifenthrin | metolachlor | chloroneb | dimethoate | prophos | p,p'-DDD |
| --- | --- | --- | --- | --- | --- | --- | --- | --- | --- | --- | --- | --- | --- | --- | --- | --- |
| deltamethrin | -0.09 | 0.16 | **0.28** | -0.05 | -0.03 | 0.06 | 0.17 | -0.23 | 0.21 | 0.22 | 0.04 | 0.02 | -0.11 | **0.24** | -0.19 | -0.06 |
| cypermethrin |  | -0.08 | 0.15 | -0.07 | -0.09 | -0.13 | 0.05 | **0.38** | 0.02 | 0.02 | -0.18 | -0.17 | -0.07 | 0.20 | 0.00 | 0.17 |
| λ-cyhalothrin |  |  | **0.32** | 0.01 | -0.04 | -0.10 | **0.25** | -0.09 | 0.16 | 0.14 | -0.07 | -0.05 | 0.06 | 0.23 | -0.07 | -0.14 |
| chlorpyrifos |  |  |  | -0.13 | 0.16 | 0.02 | 0.10 | -0.02 | 0.10 | -0.03 | -0.04 | -0.05 | 0.02 | 0.09 | -0.16 | -0.05 |
| esfenvalerate |  |  |  |  | 0.17 | 0.21 | 0.17 | -0.05 | -0.18 | 0.03 | 0.03 | -0.02 | 0.03 | -0.05 | -0.13 | -0.13 |
| p,p'-DDE |  |  |  |  |  | **0.37** | 0.16 | -0.19 | 0.14 | 0.03 | **0.29** | -0.14 | -0.19 | -0.19 | -0.02 | 0.05 |
| p,p'-DDT |  |  |  |  |  |  | 0.02 | -0.18 | 0.19 | **-0.25** | 0.01 | -0.19 | 0.00 | -0.02 | -0.12 | 0.02 |
| trans-permethrin |  |  |  |  |  |  |  | -0.01 | 0.10 | **0.81** | 0.12 | **0.34** | -0.02 | 0.17 | 0.09 | -0.10 |
| malathion/  fenitrothion |  |  |  |  |  |  |  |  | -0.08 | -0.15 | -0.10 | -0.03 | -0.13 | 0.01 | 0.12 | 0.12 |
| lindane  (γ-BHC) |  |  |  |  |  |  |  |  |  | 0.01 | 0.04 | -0.13 | -0.12 | 0.04 | 0.11 | **0.29** |
| cis-permethrin |  |  |  |  |  |  |  |  |  |  | 0.22 | **0.45** | 0.03 | 0.07 | 0.15 | -0.08 |
| bifenthrin |  |  |  |  |  |  |  |  |  |  |  | 0.23 | -0.09 | 0.10 | 0.21 | -0.06 |
| metolachlor |  |  |  |  |  |  |  |  |  |  |  |  | -0.07 | 0.17 | **0.26** | -0.05 |
| chloroneb |  |  |  |  |  |  |  |  |  |  |  |  |  | -0.06 | -0.04 | -0.04 |
| dimethoate |  |  |  |  |  |  |  |  |  |  |  |  |  |  | -0.04 | -0.04 |
| prophos |  |  |  |  |  |  |  |  |  |  |  |  |  |  |  | **0.49** |

**SI Table S4.** Active ingredients included in the 63-analyte GC-ECD method that are believed to be obsolete or discontinued for use as pesticides, or subject to the Rotterdam or Stockholm Conventions [1].

|  | **Frequency of detection in 70 wristbands** | believed to be obsolete or discontinued for use as pesticides | pesticides subject to the Rotterdam Convention | prohibited or severely restricted by the Stockholm Convention |
| --- | --- | --- | --- | --- |
| aldrin | 0 | * | * | * |
| captafol | 0 |  | * |  |
| chlordane | 0 |  | * | * |
| chlorobenzilate | 0 | * | * |  |
| chloroneb | 4 | * |  |  |
| chlorpropylate | 0 | * |  |  |
| diallate | 0 | * |  |  |
| DDT | 23 |  | * | * |
| dieldrin | 0 | * | * | * |
| endrin | 0 | * |  |  |
| endosulfan (or by-products) | 3 |  | * | * |
| heptachlor | 1 | * | * | * |
| hexachlorobenzene | 0 |  | * | * |
| HCH (mixed isomers) | 0 |  | * | * |
| isodrin | 0 | * |  |  |
| lindane | 13 |  | * | * |
| mirex | 0 | * |  |  |


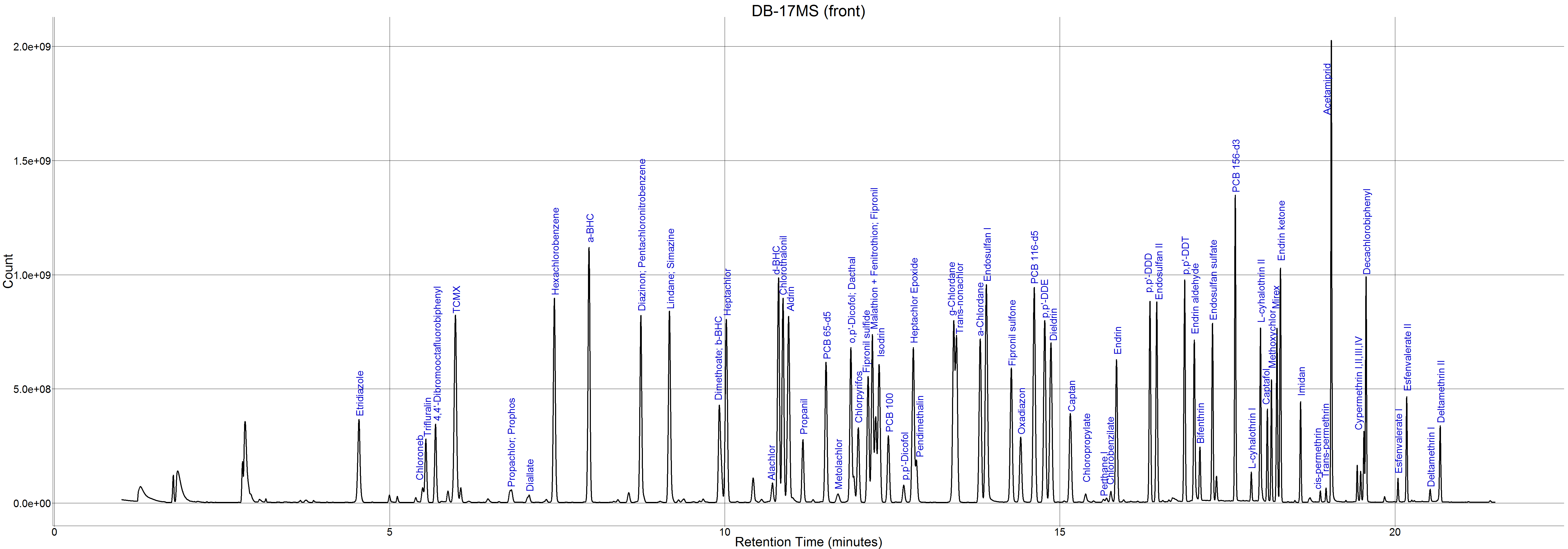

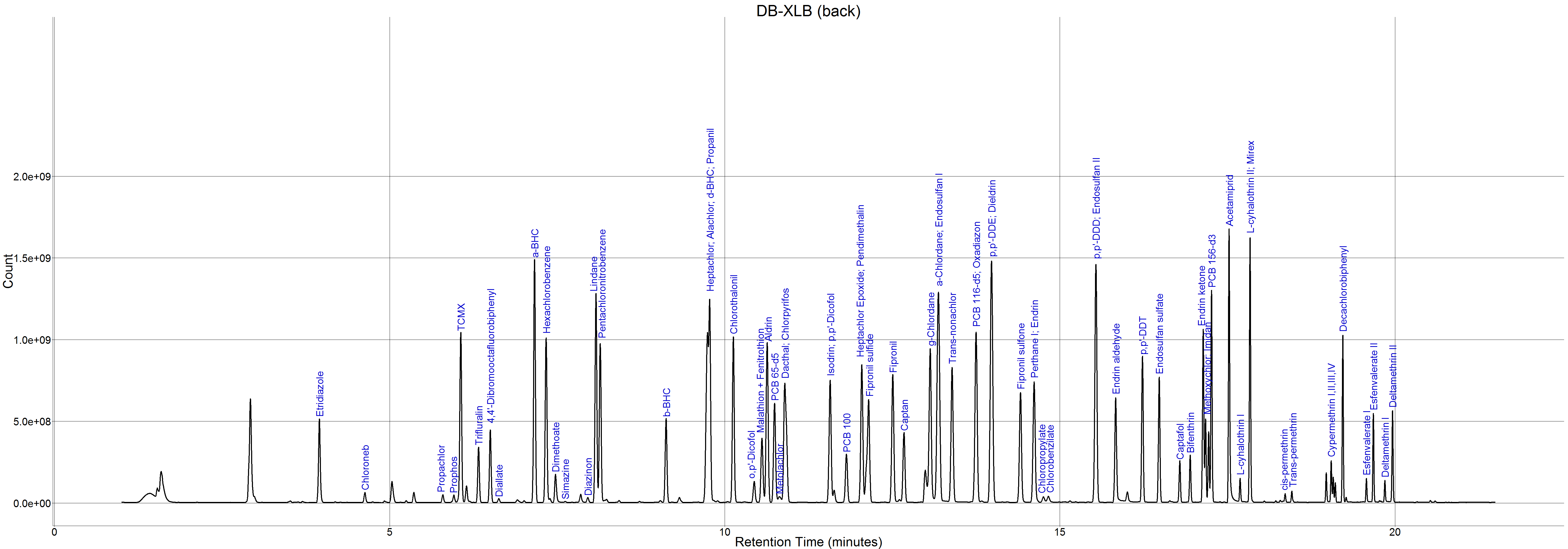


**B**

**A**

**SI Figure S1.** Example 250 μg/L standard chromatograms on 17-MS (A) and XLB (B) columns.

**
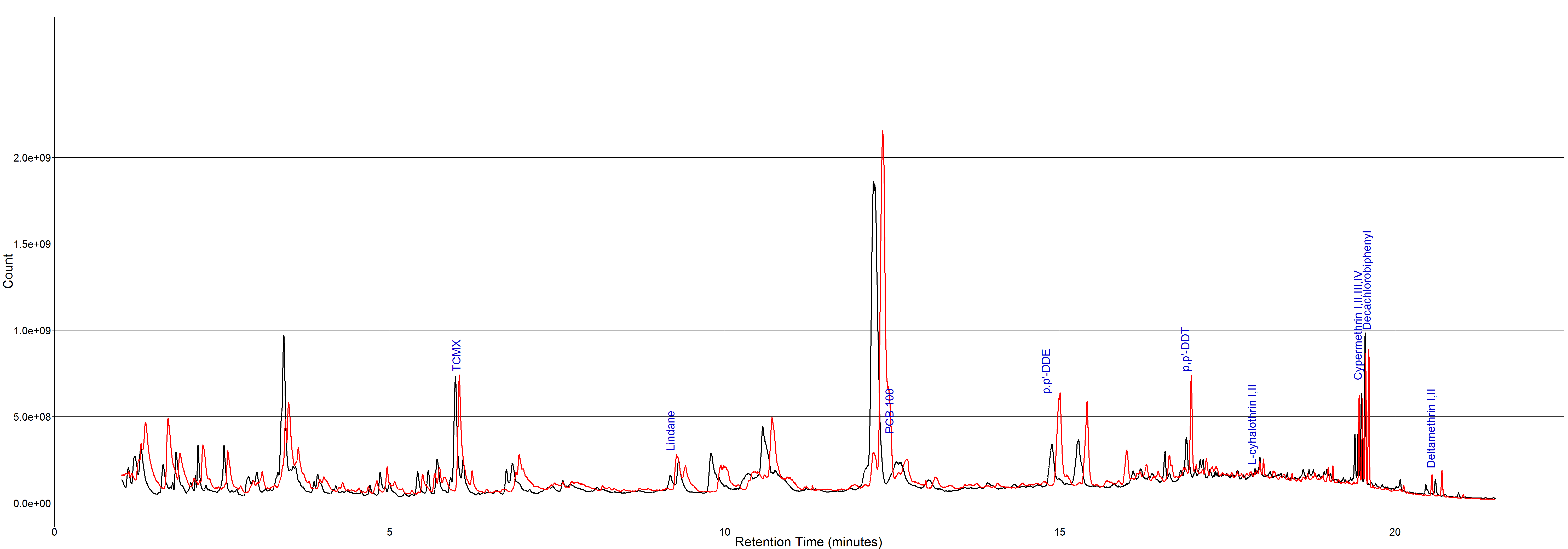
**

**A**

**
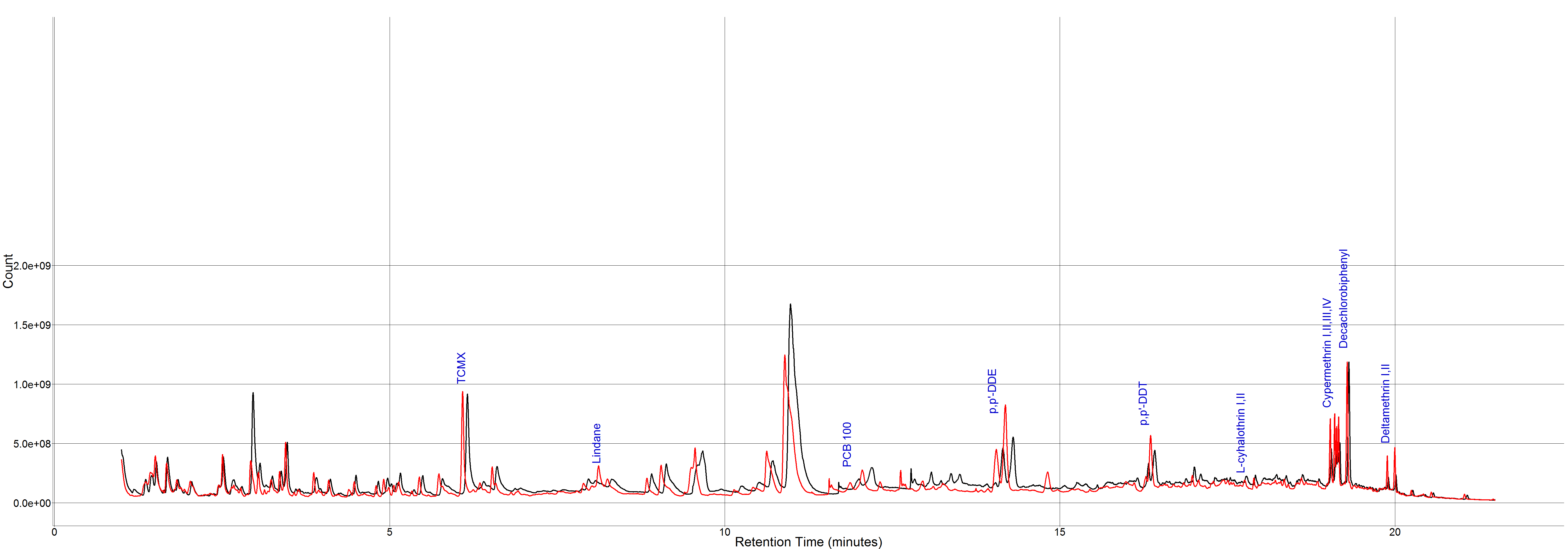
**

**B**

**SI Figure S2.** Example wristband sample extracts on 17-MS (A) and XLB (B) columns. The black chromatograms are a wristband sample extracted with TCMX, PCB 100, and decachlorobiphenyl as extraction surrogate standards. The red chromatogram is the sample extract that includes extraction surrogates and standard overspike of lindane, *p,p’*-DDE, *p,p’*- DDT, λ-cyhalothrin, cypermethrin, and deltamethrin (800-4,000 μg/L)

**
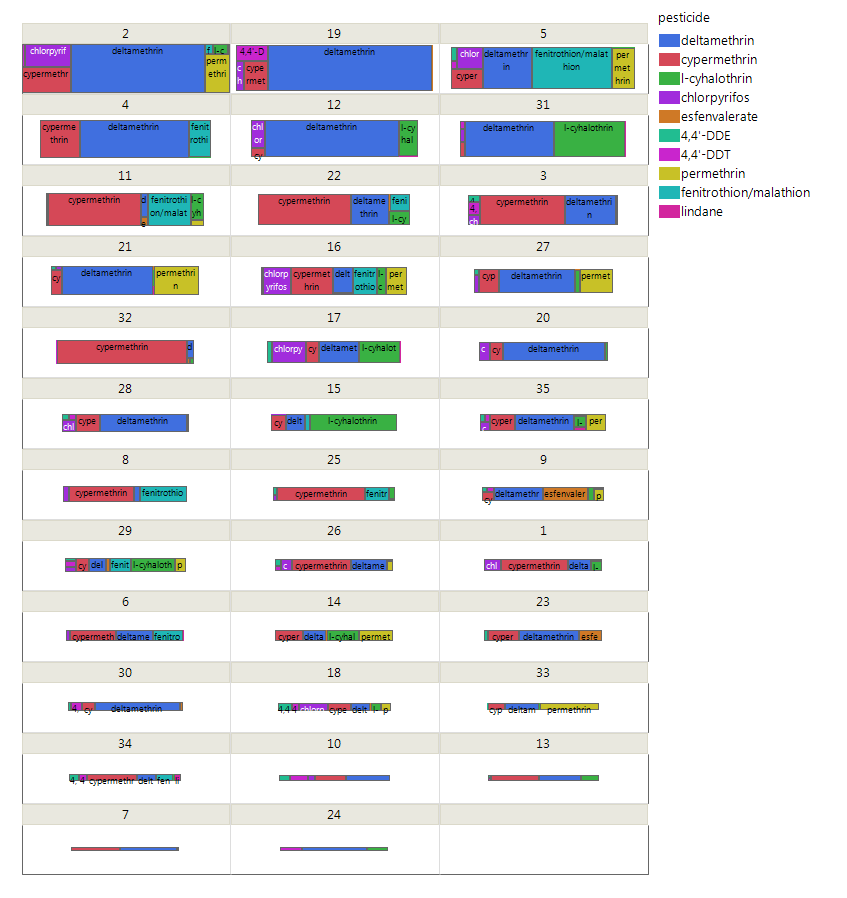
SI Figure S3.** Average concentration of 10 most-frequently detected pesticides by participant. Area is proportional to concentration of pesticides above limit of detection. Participants are ordered by total concentration.


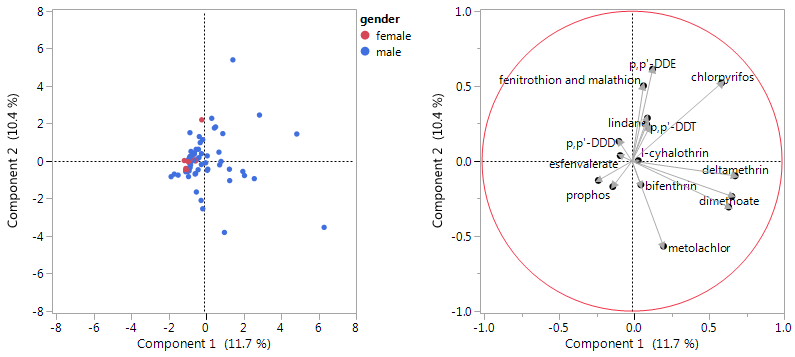
**SI Figure S4.** Principle component analysis scatterplot with participant gender designated (A) and pesticide loading plot (B).

**B**

**A**

Additional pesticides detected in comparison studies in Figure 4, main text.

Other pesticides reported in [3],but not in the present GC-ECD method: 2,4-D, acephate, atrazine, azadirachtin, bensulfuron methyl, carbofuran, copper oxychloride, diazinon, dichlorprop, glyphosate, imadacloprid, malathion, maneb, methamidophos, paraquat dichloride, profenofos, sulfur, thiophanate methyl, thiram, and triazophos.

Other pesticides reported in [4], but not in the present GC-ECD method: benzyl benzoate, diallate, N,N-diethyl-meta-toluamide, diphenylamine, ethiolate, and hexachloracyclopentadiene.

Other pesticides reported in [5], but not in the present GC-ECD method: carbaryl, carbofuran, chlorfenson, methylparathion, monocrotophos, naphthalene, pirimiphos methyl, profenofos, propoxur, sulfur, tetrafidon, and tetramethrin.

**References**

[1] World Health Organization. 2009 The WHO Recommended Classification of Pesticides by Hazard. (Geneva, Switzerland.

[2] U.S. Environmental Protection Agency. 2015 Estimation Programs Interface Suite™ for Microsoft® Windows, v 4.1.25. (Washington, DC, USA, United States Environmental Protection Agency.

[3] Jepson, P.C., Guzy, M., Blaustein, K., Sow, M., Sarr, M., Mineau, P. & Kegley, S. 2014 Measuring pesticide ecological and health risks in West African agriculture to establish an enabling environment for sustainable intensification. *Philosophical transactions of the Royal Society of London. Series B, Biological sciences* **369**, 20130491-20130491. (doi:10.1098/rstb.2013.0491).

[4] Murphy, M.W., Sanderson, W.T., Birch, M.E., Liang, F., Sanyang, E., Canteh, M., Cook, T.M. & Murphy, S.C. 2012 Type and Toxicity of Pesticides Sold for Community Vector Control Use in the Gambia. *Epidemiology Research International* **2012**, 1-6. (doi:10.1155/2012/387603).

[5] Anderson, K.A., Seck, D., Hobbie, K.A., Traore, A.N., McCartney, M.A., Ndaye, A., Forsberg, N.D., Haigh, T.A. & Sower, G.J. 2014 Passive sampling devices enable capacity building and characterization of bioavailable pesticide along the Niger, Senegal and Bani Rivers of Africa. *Philos T R Soc B* **369**, 20130110. (doi:10.1098/rstb.2013.0110).
